# Impact of Standardized Allergen-Removed *Rhus verniciflua* Stokes Extract on Advanced Adenocarcinoma of the Ampulla of Vater: A Case Series

**DOI:** 10.1155/2013/203168

**Published:** 2013-04-22

**Authors:** Woncheol Choi, Soomin An, Eunmi Kwon, Wankyu Eo, Sanghun Lee

**Affiliations:** ^1^Department of Medical Consilence, Graduate School, Jukjeon Campus, Dankook University, 152 Jukjeon-ro, Suji-gu, Yongin-si 448-701, Republic of Korea; ^2^Department of Clinical Oncology, Integrative Cancer Center, Kyung Hee University Hospital at Gangdong, Seoul, Republic of Korea; ^3^Department of Hematology and Oncology, Kyung Hee University Hospital at Gangdong, Seoul, Republic of Korea

## Abstract

*Background*. Adenocarcinoma of the ampulla of Vater (AAV) is a rare malignancy that has a better prognosis than other periampullary cancers. However, the standard treatment for patients with relapsed or metastatic AAV has not been established. We investigated the clinical feasibility of standardized allergen-removed *Rhus verniciflua* stokes (aRVS) extract for advanced or metastatic AAV. *Patients and Methods*. From July 2006 to April 2011, we retrospectively reviewed all patients with advanced AAV treated with aRVS extract alone. After applying inclusion/exclusion criteria, 12 patients were eligible for the final analysis. We assessed the progression-free survival (PFS) and overall survival (OS) of these patients during the follow-up period. *Results*. The median aRVS administration period was 147.0 days (range: 72–601 days). The best tumor responses according to Response Evaluation Criteria in Solid Tumors were as follows: two with complete response, two with stable disease, and eight with progressive disease. The median OS was 15.1 months (range: 4.9–25.1 months), and the median PFS was 3.0 months (range: 1.6–11.4 months). Adverse reactions to the aRVS treatment were mostly mild and self-limiting. *Conclusions*. Prolonged survival was observed in patients with advanced AAV under the treatment of standardized aRVS extract without significant adverse effects.

## 1. Background

Adenocarcinoma of the ampulla of Vater (AAV) is a rare cancer representing about 6% of all periampullary tumors and only 0.2% of all gastrointestinal cancers [1, 2]. From 1973 to 2005, 5,625 cases of AAV were reported in the USA, and the frequency of the disease has been increasing [3]. Furthermore, Asian-Pacific people have a higher incidence of this cancer, whereas African American men have a slightly lower incidence in the USA [3, 4]. 

The prognosis of AAV is better with a higher resectability rate compared to other periampullary malignancies including the pancreatic or the distal bile duct cancers because even small tumors of the ampulla generally produce obstructive jaundice earlier than tumors in other locations [4–6]. On the other hand, patients who relapse or present with metastatic AAV have a poor prognosis with a two-year overall survival rate of about 10% [3]. There is no standard chemotherapeutic regimen for advanced AAV patients and no standard adjuvant therapy after curative pancreaticoduodenectomy. Further, a considerable number of patients could not undergo aggressive chemotherapy or radiation therapy because of poor performance status or adverse effect [4]. 

*Rhus verniciflua* Stokes (RVS) of the Anacardiaceae family, commonly known as the lacquer tree, was traditionally used for treating diseases of the digestive system, including tumors, in Korea and China in the 15th century [7, 8]. Several reports have been published on the clinical efficacy of RVS in stabilizing cancer progression or inducing tumor regression [9–14]. Many experimental studies have recently shown that the flavonoids from RVS or the whole extract have antiproliferative and apoptotic activities in human cancer cell lines [7, 8, 15–17]. Here, we report on the outcomes of using a standardized extract from RVS (aRVS) without conventional therapy regimens in patients with advanced AAV.

## 2. Patients and Methods

### 2.1. Patients

All patients had advanced AAV and were treated with the standardized aRVS extract at the Integrative Cancer Center, Kyung Hee University Hospital at Gangdong (Seoul, Republic of Korea) between June 1, 2006 and April 30, 2011. We selected patients based on the following eligibility criteria: (1) histologically confirmed AAV, (2) radiological findings showing nonresectability of the tumor that included evidence of distant metastasis, and (3) more than a day of standardized aRVS extract administration. Exclusion criteria included response evaluation not available after aRVS treatment and concurrent conventional treatment including chemotherapy or radiotherapy during aRVS treatment. All patients signed a written informed consent form. Medical records were retrospectively reviewed with particular attention to the initial history and physical examination, histopathologic findings, operative and postoperative treatments, and followup. This study was approved by the institutional review board of the Kyung Hee University Hospital at Gangdong (KHNMC-OH-IRB 2011-12).

### 2.2. Standardized Extract of aRVS and Treatment Course

The clinical application of RVS has been limited because of an allergenic component, urushiol, which causes severe contact dermatitis in sensitive individuals [18–20]. Therefore, urushiol, a mixture of several derivatives of catechol, must be removed from RVS prior to its pharmaceutical use. A standardized extract of allergen-removed RVS (aRVS) was manufactured based on thorough historical research (fustin > 13.0%, fisetin > 2.0%, urushiol not detected). The daily oral administration of 1350 mg (one 450-mg capsule, three times a day) of aRVS extract was prescribed.

### 2.3. Evaluation of Efficacy and Safety

We identified 15 patients with advanced AAV who were consecutively treated with aRVS. Three patients were excluded because of a lack of response evaluation (*N* = 2) and concurrent radiotherapy (*N* = 1).

We assessed the treatment outcomes of progression-free survival (PFS), overall survival (OS), and toxicities. Progression of radiological findings was determined according to Response Evaluation Criteria in Solid Tumors (RECIST) 1.1. Disease status was radiologically checked every two to three months after aRVS treatment. OS was defined as the period from the date of the start of aRVS treatment until death from any cause. We verified the time of death using official Korean National Health Insurance records on March 15, 2012. Both PFS and OS were estimated using the Kaplan-Meier method. Safety was assessed in terms of toxicity and assigned a severity grade ranging from 1 to 4 based on the Common Terminology Criteria for Adverse Events (CTCAE), version 4.0.

## 3. Results

### 3.1. Clinical Characteristics

The baseline characteristics of the patients are presented in Table 1. The median age was 52 years (range: 36–73 years), with a low BMI (median BMI: 20.3; range: 14.6–25.2). Ten (83.3%) patients had undergone surgical resection of their primary tumor, and, of those, two (20.0%) had received adjuvant treatment. 

Only three patients (25.0%) were chemotherapy naïve because of advanced age, poor performance status, anxiety about the toxicity of chemotherapy, or preference for herbal medicine. Before starting aRVS treatment, nine patients (75.0%) had received prior palliative chemotherapy, and three of those patients (33.3%) had received second chemotherapy regimens. All patients had progressive disease during or after prior chemotherapy except for one patient (ID 9).

### 3.2. Safety and Treatment Outcomes of the aRVS Extract

On March 15, 2012, nine patients had expired, and the remaining three patients were living. The median aRVS administration period was 147.0 days (range: 72–601 days). Overall, treatment was well tolerated, even in patients with a worse performance status. Although hematologic toxicity related to aRVS treatment was not observed, nonhematologic adverse effects were reported. One case of gastritis (Gr 2) and two cases of pruritus (Gr 1) were each observed in three patients. The reported gastritis developed after surgery, and the symptoms waxed and waned. The symptom of pruritis spontaneously diminished without reducing the dosage of aRVS. Patients discontinued aRVS treatment because of disease progression, not because of adverse effects of aRVS.

The best tumor responses based on RECIST, PFS, and OS are summarized in Table 2. The best responses among the 12 patients were complete response in 2 cases (ID 8 and 11), stable disease in 2 cases (ID 3 and 6), and progressive disease in 8 cases. The median OS was 15.1 months (range: 4.9–25.1 months). The median PFS was 3.0 months (range: 1.6–11.4 months).

**Figure 1:**
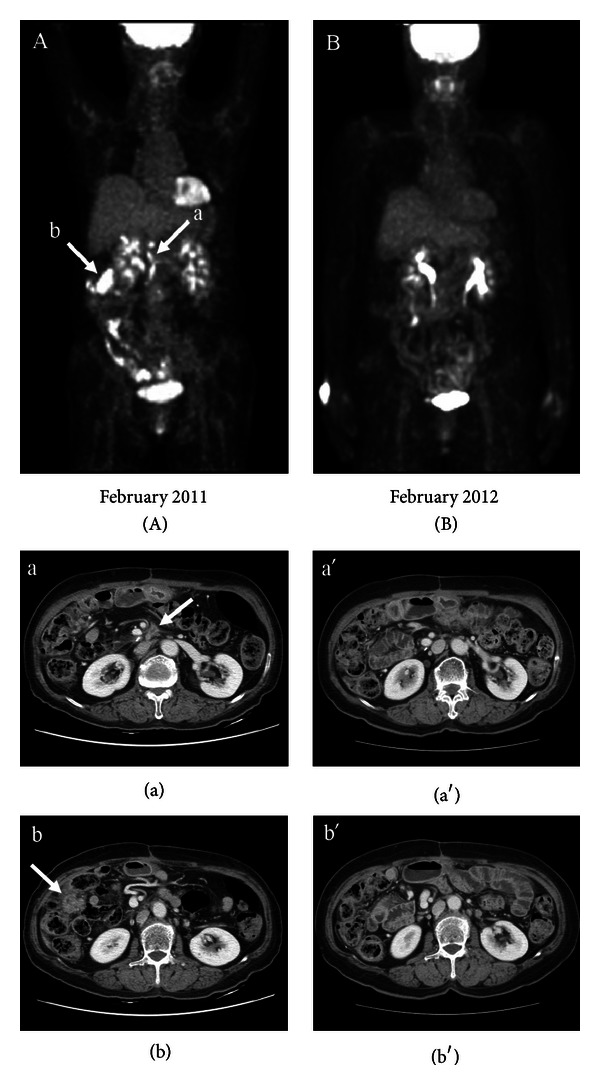
Multiple peritoneal metastases after surgery were confirmed by a PET-CT whole-body scan in February 2011 (A). The irregular speculated soft tissue mass in the proximal superior mesenteric artery lesion (20 mm; (a)) and the right side mesentery mass (32 mm; (b)) disappeared in the recent CT scans ((a′) and (b′)) after only aRVS treatment. All the metastases disappeared in the recent PET-CT scans in February 2012 after only aRVS treatment (B).

Patient 11 is 73 years old and was diagnosed with adenocarcinoma with invasive pancreas (pT3N0 M0; IIA) in June 2010, following an abnormal presentation during a routine health examination. She underwent pylorus preserving pancreatoduodenectomy (PPPD). Follow-up CT scans in February 2011 revealed newly developed peritoneal seeding and local tumor recurrence around the PPPD site. PET-CT whole body scans confirmed multiple peritoneal metastases (Figure 1(A)). The patient and her family refused palliative chemotherapy because of old age and poor performance status. For these reasons, the patient visited our hospital to receive alternative therapy. The treatment plan did not include any orthodox therapies such as surgical operation, chemotherapy, or radiation therapy. Only aRVS was administrated since March 2011. Follow-up CT scans in July 2011 showed a decrease (12 mm ← 20 mm) in the recurrent mass in the superior mesenteric artery lesion and disappearance of the right side mesenteric mass (32 mm) and peritoneal nodules. Recent PET-CT scans in February 2012 revealed complete disappearance of her AAV (Figure 1(B)). She is currently doing well.

**Figure 2:**
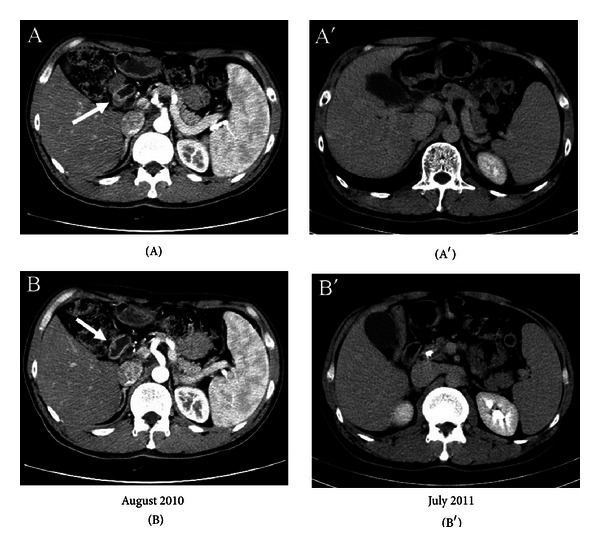
An abdominal CT scan in August 2010 revealed a mass in the ampulla of Vater legion ((A) & (B)). After only aRVS treatment, a recent abdominal CT scan in July 2011 showed that no definite mass is seen in the region of the ampulla of Vater nor is there any definite evidence for metastatic mass ((A′) and (B′)).

Patient 8, a 35-year-old patient with liver metastasis from AAV, underwent six cycles of treatment with oxaliplatin/capecitabine and achieved complete response of the liver mass and stable disease in the ampullary mass. However, the patient stopped his treatment because of thrombocytopenia and neuropathy from chemotherapy. Instead, he has received aRVS treatment since August 2010. After three months of only aRVS treatment, the ampullary mass completely disappeared. Follow-up CT scans in July 2011 showed no evidence of tumor recurrence (Figure 2). The patient is currently doing well with refusing further radiological examination.

**Figure 3:**
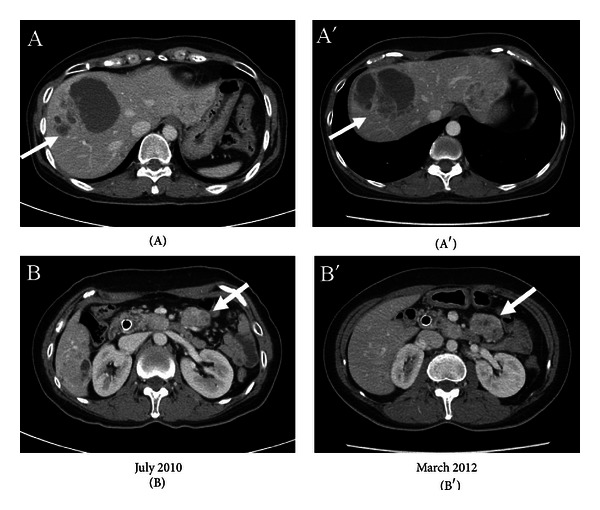
An abdominal CT scan in July 2010 revealed a necrotic metastatic lesion in the right lobe of liver and a metastatic mass in mesentery LUQ ((A) & (B)). After only aRVS treatment, a recent abdominal CT scan in March 2012 showed slight increase in size of metastatic masses over 20 months ((A′) and (B′)).

Patient 6, a 45-year-old patient was diagnosed with liver, pancreas, and peritoneal metastases from AAV in March 2010. She received three cycles of cisplatin/5FU/epirubicin, and follow-up CT scans in July 2010 unfortunately showed the progression of metastatic masses in liver. Continuous palliative regimen such as gemcitabine-based chemotherapy was recommended, but she strongly refused it because she experienced adverse effects and poor response from chemotherapy. She has received aRVS treatment as alternative therapy since July 2010 (Figures 3(A) and 3(B)). In spite of the tumor progression in June 2011, she insisted aRVS treatment because her disease slowly progressed and there were no adverse effects from aRVS treatment. The recent CT scans in March 2012 showed no significant change, indicating stable disease compared to the CT scans in June 2011 (Figures 3(A′) and 3(B′)). The patient is fully active doing well without signs or symptoms of disease.

## 4. Discussion

AAV represents a rare disease, and only a few prospective studies on the palliative treatment of AAV have been published [21, 22]. Moreover, previous studies were small and focused on small bowel adenocarcinoma having significantly better OS than AAV. To the best of our knowledge, there is no prospective randomized controlled study evaluating the benefit of palliative chemotherapy against AAV. Therefore, patients with advanced AAV are obliged to receive chemotherapy based on their physician's preference [4].

Recently, two clinical reports were published on the efficacy of chemotherapy in patients with advanced relapsed/metastatic AAV. A phase II trial of capecitabine plus oxaliplatin in 25 patients with metastatic adenocarcinoma of the small bowel and ampullary origin reported a median time to progression (TTP) of 6.6 months and a median OS of 15.5 months [21]. However, the study included patients with small bowel origin rather than AAV. The response rate was actually 33% in AAV cases, which is poor, compared to a rate of 61% in cases with small bowel origin. The retrospective data from another Korean hospital showed that first-line cisplatin-based combination chemotherapy in AAV resulted in a median TTP of 4.9 months and a median OS of 12.5 months [23]. These results are consistent with the outcomes of the current study because it was a retrospective review of patients of the same ethnicity with only AAV, not small bowel adenocarcinoma.

The median OS of 15.1 months in our study is comparable to the findings based from the other Korean hospital, although our sample size was smaller (12 patients versus 29 patients). It should be noted that most patients (75.0%) in our study were refractory to the first or second regimen of chemotherapy and the median time was 8.4 months from the day of advanced or metastatic stage diagnosis to the initiation of aRVS treatment (Tables 1 and 2). The more conventional treatments were not available and recommended for them by oncologist because of disease progression or adverse effect. Nonetheless, there were two cases (ID 8 and 11) out of 12 patients showing complete response after only aRVS treatment, while the front-line cisplatin-based chemotherapy did not result in a single case of complete response out of 29 patients.

The aRVS treatment was safe with over one year of administration (ID 4, 6, and 8), and there were no aRVS discontinuations related to adverse effects. Hematologic toxicity was not reported, and only minor grade nonhematologic adverse effects were reported in three patients. The results also suggest that aRVS treatment could improve OS, in spite of a median PFS of only 3.0 months, which is supported by the outcomes of patients in this study (ID 2, 3, 4, 5, and 6). Therefore, the aRVS treatment should not be evaluated as the cytotoxic agents focusing on tumor response.

Biological studies on AAV have been difficult because there are only a few established cell lines and histological subtypes are mixed with either pancreatobiliary or intestinal differentiation [24, 25]. Therefore, there is a need for a better understanding of the biology of AAV and the identification of potential prognostic factors and clinically relevant molecular targets for therapy. Up to now, several clinical reports have demonstrated that the expression of death-promoting proteins such as Bax is highly correlated with survival in patients with radically resected AAV, and cyclooxygenase-2 (COX-2) expression is highly elevated in patients with AAV and strongly associated with vascular endothelial growth factor (VEGF) expression [26–28].

The flavonoids are known to have significant anticancer properties as they induce apoptosis and inhibit COX-2 [29]. A purified flavonoid fraction prepared from RVS has been shown to induce apoptosis in some cancer cell lines, while sparing normal cells [7, 16]. A phenolic-rich fraction from RVS was reported to suppress inflammatory response via the NF-*κ*B and JNK pathway [30]. Sulfuretin (3′,4′,6-trihydroxyaurone) isolated from RVS inhibits COX-2 as well as proinflammatory cytokine expression via the downregulation of NF-*κ*B [31]. Fisetin (3,7,30,40-tetrahydroxyflavone) from RVS was also found to have apoptotic, antiproliferative, and anti-invasive effects by downregulation of the NF-*κ*B signaling pathway in chemo-resistant human pancreatic cancer cells [32]. Butein (3,4,2′,4′-tetrahydroxychalone) has also been shown to downregulate COX-2 expression in cancer cells and suppress cancer cell micrometastasis by inhibiting fibroblast formation [33, 34]. It has been experimentally demonstrated that the standardized aRVS extract has antiangiogenic activities by inhibiting VEGF and an inhibitory effect on matrix metalloproteinase-2 (MMP-2) and MMP-9 activities in a human fibrosarcoma cell line [35, 36]. The single component such as fustin or fisetin shows poor results compared to the whole extract. Though the mechanism of action is not clear, the multifactorial synergistic interactions among unknown compounds in RVS are thought to block AAV progression with major biologically active components such as sulfuretin, fisetin, and/or butein. 

Even though this study is limited by the rarity of the disease, our results indicate that aRVS extract is beneficial in tumor response to some patients (ID 6, 8, and 11) previously described and could be applied as a salvage regimen or adjuvant agent against AAV in the selected patients for whom chemotherapy is not feasible. Therefore, we have tried to find several markers in common among responders, based on traditional medicine and modern science in the view of personalized medicine. For example, the alterations of cytokines-related immune system or metabolites in urine and plasma are investigated before and after treatment. We also hope that this clinical outcome would stimulate different investigation in natural products from conventional drug discovery and development based on cytotoxicity [14].

## Figures and Tables

**Table 1:** Demographic and clinical characteristics of all patients (*N* = 12).

Patient number	Gender	Age	BMI	Diagnosis day	Initial stage*	Prior surgery	Differentiation	Prior adjuvant treatment	Day of advanced stage diagnosis	Metastasis region	Chemotherapy regimen	Response
1	Female	65	23.4	2005-07-15	I	PPPD	NA		2006-07-15	Liver	(1) Clinical trial 4 cycles (2) Chemotherapy 2 cycles not reported	PD
2	Female	61	20.6	2005-04-25	IIB	PPPD	Well		2005-12-30	Metastatic LN	(1) Oral FU/CDDP 15 cycles (2) Gemcitabine 4 cycles	PD
3	Female	58	25.2	2006-08-07	IV	PPPD (R2)	Moderate			Lung, LN	(1) Oral FU with concurrent RT 1 cycles	PD
4	Female	36	18.5	2008-05-20	IIB	PPPD	Moderate		2008-12-15	Lung, bone, LN	(1) Gemcitabine/CDDP 8 cycles (2) FOLFOX 2 cycles	PD
5	Male	66	24.7	2006-12-18	IB	PPPD	NA	IV FU with concurrent RT	2007-08-21	Liver	(1) Oral FU/CDDP 21 cycles	PD
6	Female	45	20.1	2010-03-31	IV		Poor			Liver	(1) IV FU/CDDP/EPS 3 cycles	PD
7	Male	68	17.9	2009-08-04	IIB	PPPD	Moderate		2009-12-02	Lung, LN	(1) Oral FU/CDDP 8 cycles	PD
8	Male	41	20.8	2010-03-17	IV		Well			Liver	(1) Oral FU/Oxaliplatin 6 cycles	SD
9	Male	46	19.7	2009-08-05	IIB	PPPD	NA		2010-12-01	Peritoneal seeding	Chemotherapy refused because of adverse effects	
10	Female	41	14.6	2009-09-22	IIB	PPPD	Moderate	Oral FU 2 cycles	2010-02-06	Lung, liver	Chemotherapy refused because of adverse effects	
11	Female	73	22.6	2010-06-14	IIA	PPPD	Well		2011-02-09	Peritoneal seeding	Chemotherapy refused because of old age	
12	Male	37	19.3	2008-11-15	IIB	PPPD	Moderate		2009-10-15	Mesenteric LN	(1) Oral FU with concurrent RT 1 cycles	PD

*Staging is based on the seventh edition of the *TNM Classification of Malignant Tumors*.

BMI: body mass index, LN: lymph node, NA: not available, PD: progressive disease, PPPD: pylorus preserving pancreaticoduodenectomy, RT: radiotherapy, and SD: stable disease.

**Table 2:** Patient response to aRVS extract treatment.

Patient number	Initial day of aRVS treatment*	Time from advanced stage diagnosis to aRVS treatment (month)	aRVS treatment duration (month)	Best response RECIST	Progression-free survival (month)	Overall survival (month)
1	2007-02-28	7.6	6.0	PD	2.7	6.7
2	2007-06-01	17.3	4.6	PD	1.6	15.1
3	2007-06-18	10.5	4.7	SD	4.1	25.1
4	2009-09-09	8.9	15.5	PD	1.9	16.9
5	2010-07-14	35.3	5.1	PD	3.3	14.2
6	2010-07-23	3.8	20.0	SD	11.2	20.0 (alive)
7	2010-07-27	7.9	3.5	PD	1.7	10.3
8	2010-08-11	4.9	13.8	CR	11.3 (+)	19.4 (alive)
9	2011-01-14	0.5	2.4	PD	2.7	4.9
10	2011-01-26	11.8	4.0	PD	2.2	11.8
11	2011-03-16	1.2	10.7	CR	11.4 (+)	12.2 (alive)
12	2011-03-30	17.7	2.6	PD	3.3	6.7

*The standardized allergen-removed *Rhus verniciflua* Stokes (aRVS) extract (1350 mg) was orally administered daily.

CR: complete response, PD: progressive disease, PR: partial response, and SD: stable disease.
